# Advancing archaeological sedimentary lipid biomarker analysis: A review of recent developments and methodological guidelines

**DOI:** 10.1016/j.isci.2025.112064

**Published:** 2025-02-25

**Authors:** Carolina Mallol, Natalia Égüez, Margarita Jambrina-Enríquez, Antonio V. Herrera-Herrera

**Affiliations:** 1 Instituto Universitario de Bio-Orgánica Antonio González, Universidad de La Laguna, Avda. Astrofísico Fco. Sánchez, 2, 38206 San Cristóbal de La Laguna, Spain; 2 Departamento de Geografía e Historia, UDI Prehistoria, Arqueología e Historia Antigua, Facultad de Humanidades, Universidad de La Laguna, 38206 Tenerife, Spain; 3 Department of Anthropology, University of California, Davis, One Shields Avenue, Davis, CA 95616, USA; 4 Departamento de Ciencias de la Vida y de la Tierra, Instituto de Productos Naturales y Agrobiología, Consejo Superior de Investigaciones Científicas (IPNA-CSIC), 38206 Tenerife, Spain; 5 Departamento de Biología Animal, Edafología y Geología, Facultad de Ciencias, Universidad de La Laguna, 38206 Tenerife, Spain; 6 Departamento de Química, Unidad Departamental de Química Analítica, Facultad de Ciencias, Universidad de La Laguna, Avenida Astrofísico Francisco Sánchez, s/n, 38206 San Cristóbal de La Laguna, Spain

**Keywords:** Biochemical analysis, Paleobiochemistry, Archeology

## Abstract

This review targets archaeological scientists and geoarchaeologists, examining the current state of lipid biomarker analysis in archaeological sediments—a growing field. Lipid compounds and their stable isotope ratios serve as valuable proxies for reconstructing past climates, vegetation, freshwater availability, human-environment interactions, diet, technology, and subsistence practices. The paper reviews experimental, archaeological, and ethnoarchaeological studies that apply lipid biomarkers to archaeological sedimentary deposits, contributing to paleoenvironmental research and insights into past human behavior. Key topics include fecal biomarkers, revealing diet and subsistence, and pyrogenic biomarkers, shedding light on fire technology and cooking traditions. Methodological guidelines are provided, covering sample collection, lipid extraction, pretreatment, and compound detection. Challenges include standardizing protocols, integrating new biomarkers, microcontextual approaches, and adopting advanced analytical techniques. Advancing lipid biomarker analysis promises to enhance interdisciplinary research and deepen our understanding of archaeological contexts and human-environment dynamics.

## Graphical abstract

**Figure:**
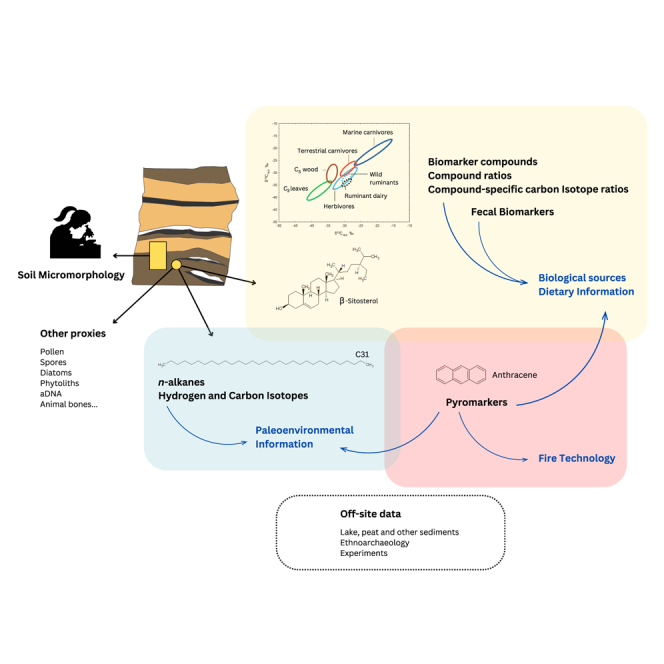

## Introduction

Lipids are the hydrophobic organic molecular compounds of fats, oils, and waxes. They are produced by living organisms for specific metabolic functions. For example, epicuticular leaf waxes protect plant leaves from microbial infections, UV radiation and water loss and saturated fatty acids, such as palmitic and stearic acids are the building blocks of triglycerides, phospholipids, and adipose tissue.^1^ Overall, lipids play crucial roles in energy metabolism, structural integrity and physiological processes of all living organisms. Their degree of hydrophobicity or polarity is determined by the functional groups present in their chemical structure, by which they can be classified from less to more polar as: non-linear and linear hydrocarbons, aromatics, esters, aldehydes, ketones, alcohols, and carboxylic acids (among which alkanoic or fatty acids stand out). Lipid biomarkers or lipid biological molecular markers are specific lipid compounds whose chemical structure is functionally and/or genetically determined such that particular metabolic pathways or classes of living organisms can be traced.^2^^,^^3^^,^^4^

Lipid biomarker analysis started to have an impact in the archaeological discipline around the mid twentieth century, with the technical advance of instrumental analytical chemistry and the development and optimization of chromatography, particularly liquid chromatography (LC) and gas chromatography (GC), and their combination with flame ionization detectors (FID) and mass spectrometry.^5^ Since then, lipid biomarker analysis of archaeological remains using different lipid detection techniques and in combination with compound-specific isotope analysis (CSIA), particularly focused on hydrogen and carbon isotope ratios, has contributed a rich body of knowledge on the organic archaeological record and its preservation potential.^6^^,^^7^^,^^8^^,^^9^ The field of lipid biomarkers in archaeology has focused on the analysis of lipid residues found on objects, especially in pottery,^10^^,^^11^^,^^12^^,^^13^^,^^14^^,^^15^^,^^16^^,^^17^^,^^18^^,^^19^^,^^20^^,^^21^^,^^22^ but also in coprolites and bone,^23^^,^^24^^,^^25^^,^^26^ stone objects,^27^^,^^28^^,^^29^ floors,^30^^,^^31^^,^^32^^,^^33^ and synthetic pigments.^34^^,^^35^

**Figure 1:**
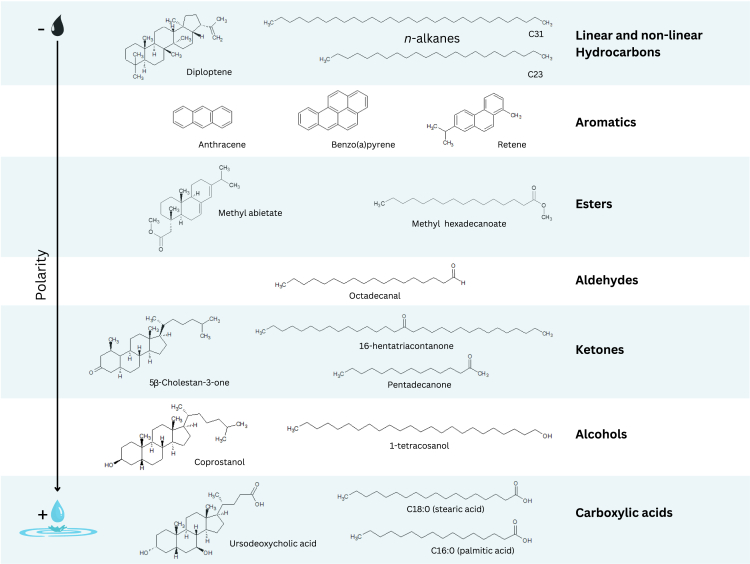
Examples of lipid compounds of different polarity that have been found in archaeological sedimentary contexts These may represent different biological sources whose presence in archaeological contexts is informative toward understanding the formation of the deposit or specific behaviors or human activities. For example, nonlinear hydrocarbons such as the hopanoid diploptene might indicate presence of soil bacteria, while the presence of certain aromatic compounds and long-chain ketones may point toward combustion-related activity. For instance, retene forms through the chemical degradation of diterpenes found in conifer resins, particularly abietic acid, and has been associated with pine burning activity. This compound may be found in association with methyl abietate, an ester derived from abietic acid. Aldehydes, such as octadecanal, may be used as biomarkers of contexts with lipid degradation, as they derive from oxidative breakdown of fatty acids. Finally, alcohols and carboxylic acids hold great potential for biomarker analysis, comprising a diversity of compounds that may allow us to assess the contribution of terrestrial vs. aquatic macrophytes and algae, or to trace the presence of specific plants and animal families based on their chain length and relative presence.

A predominant proportion of archaeological contexts is sedimentary, and archaeological sediment conceals valuable information about humans and their environment. Lipids, in particular, have a high archaeological preservation potential due to their hydrophobic nature, and examples of different kinds of lipid compounds have been found in archaeological sedimentary deposits (Figure 1). Such biomarker compounds can provide clues about organic matter associated with past human contexts. However, lipid biomarker analysis in archaeological sediment is a relatively recent pursuit, starting to gain presence in the archaeological literature in the past few decades. Due to the tradition and knowledge base of the geoarchaeological discipline^36^ most of the techniques provide information on elemental and mineral sedimentary components, or on biological components at the microscopic scale, such as pollen, phytoliths, charcoal, and other plant remains.^37^ At the molecular scale, spectroscopic techniques based on infrared and Raman radiation traditionally provided biomolecular information by identifying organic functional groups, but this information is insufficient for sourcing organic residues contained in the sedimentary matrix. Yet, most of the original human contexts we seek to approach were organic, including the surrounding vegetation, food, clothing, bedding, and possibly most of the objects made and used in daily activities of past human groups. The mentioned spectroscopic techniques are of little use when attempting to analyze complex mixes of variably degraded organic molecules with a multitude of functional groups interacting with each other. In the present state-of-the-art, increasing accessibility and affordability of biogeochemical techniques and instrumentation and the bioarchaeological revolution^38^ have radically changed this reality, with stable isotope analysis, genetics and proteomics applied to sedimentary contexts fully incorporated into archaeological research agendas. In the case of lipids, geoarchaeologists now apply geochemical analytical techniques grounded on the pioneering works of biogeologists and organic geochemists who explored the preservation potential of lipid organic molecules and their stable isotope ratios in sediments of geological age^2^^,^^3^^,^^4^^,^^39^ and as far back as three billion years ago.^40^ This work is contributing valuable paleoenvironmental, technological, paleodietary, and chronometric data.

In this paper, we present a state-of-the-art and narrative review to explore the current state of lipid biomarker analysis in archaeological sediments, which are proving to be rich sources of lipid residues that are well-preserved and in their primary positions and hold potential to inform us about past climate and vegetation, and past human diet, technology, domestic life and subsistence practices. There are published reviews showing the potential of lipid biomarkers present in non-archaeological peat, lake and coastal sediments to inform about human impact on the landscape.^41^^,^^42^ Here, we provide a critical overview of published experimental, archaeological and ethnoarchaeological studies of lipid biomarkers present in *archaeological* sediments, with emphasis on the analytical problems that arise from the complex, mixed, time-averaged nature of archaeological sedimentary deposits and an outlook to possible future directions of this line of research. Given that the application of lipid analysis in such contexts is still relatively meagre, there are a limited number of case studies available relative to other disciplines currently applying lipid biomarker analysis, such as climate science, geobiology, or geochemistry. Consequently, we adopted a narrative review approach to capture and synthesize the most recent and relevant examples within this developing area, gathering case studies from interdisciplinary scientific literature that encompass diverse sedimentary environments, geographic regions, and time periods to ensure a comprehensive and representative overview. Our aim is to highlight the most representative studies that demonstrate the current capabilities and challenges of lipid analysis in archaeological sediments. We believe this approach provides a comprehensive overview of the existing research and serves to identify key areas for future study as the field continues to evolve. The paper is organized into three thematic sections, selected for their prevalence within the recent literature: (1) paleoenvironmental research, focusing on the widespread studies of *n*-alkane records and their carbon and hydrogen isotope ratios, (2) research into past human behavior, including diet, settlement patterns and fire use through studies of plant and animal taxonomy using biomarker compounds, ratios between different compounds, and ratios between their stable carbon isotopes, (3) methodological aspects of archaeological sediment lipid analysis, including a critical overview of common sample collection, lipid extraction, lipid extract pretreatment, and lipid compound detection and identification methods. The aim of this review is to serve as a synthetic introduction and source of relevant literature on the study of biomarkers in archaeological sediment for archaeological scientists, geoarchaeologists, and other researchers who wish to explore this domain.

## Paleoenvironmental biomarkers in archaeological sedimentary contexts

### Plant types, environmental conditions, and climatic fluctuations

Obtaining data on the climate and vegetation associated with particular archaeological contexts is crucial for behavioral interpretation. Sedimentary molecular lipid residues of plants, fungi, and bacteria and their hydrogen and carbon isotopes have been widely investigated. Hydrocarbons or *n*-alkanes and their hydrogen and carbon isotopes have been a target in paleoenvironmental sciences and organic geochemistry since the pioneering work of Eglinton and colleagues, who showed that the epicuticular leaf waxes of different plant groups have significant differences in their *n*-alkane configuration.^43^ The overwhelming presence of *n-*alkanes in soils and sediments makes them reliable proxies of paleoenvironmental conditions and they are widely investigated in non-archaeological peats, paleosols and lacustrine/fluviolacustrine sedimentary sequences, including those of paleolakes associated with early hominin sites^44^^,^^45^^,^^46^^,^^47^^,^^48^ and upper Pleistocene settings.^49^ There are also other lipid biomarkers informative of climatic or hydrological conditions that are sought in archaeological sediments, such as hopanoids, triterpenes, or branched glycerol dialkyl glycerol tetraethers (brGDGTs), which have shown to be useful in paleoenvironmental investigations of archaeological and non-archaeological sediments.^50^^,^^51^^,^^52^^,^^53^^,^^54^^,^^55^^,^^56^^,^^57^^,^^58^^,^^59^ For a recent review of biomarkers and paleoenvironmental research see McClymont et al., 2023.^60^

The *n*-alkanes, which are structurally the simplest and most resistant type of lipid compounds, constitute the basic building blocks of other more complex lipid compounds found in all living organisms, and may derive from the breakdown of a great diversity of complex lipids. They are particularly abundant in epicuticular leaf waxes, which show taxonomic variability in their *n*-alkane composition so that different plant groups or families can be discriminated. Generally, leaf waxes are made up of many linear, saturated carbon-hydrogen molecules (chains) and differ in their prevalence of chains with a specific number of carbon atoms by plant type. For example, herbaceous and gymnosperm leaf waxes typically contain long-chain *n*-alkane chains dominated by C_31_ and C_33_, while the leaf wax *n*-alkanes of woody plants are dominated by C_27_ and C_29_.^61^ However, interpreting *n-*alkane data can be difficult and requires caution and the use of complementary ratios^62^ and other biomarkers, including di- and tri-terpenoids, such as hopanoids.^63^ The average carbon chain length (ACL) and a series of other ratios such as the carbon preference index (CPI), the pristane/phytane ratio or the ratio to determine the proportion of aquatic plants (Paq) are commonly used to assess the source and preservation state of sedimentary *n*-alkanes.^6^^,^^64^^,^^65^^,^^66^^,^^67^^,^^68^ These ratios are calculated based on different molecular ranges or quantified differently across studies. It is important to note that achieving a more harmonious interpretation of these ratios requires consideration of current issues related to the difficulty of comparing them across different investigations.^69^

With their simple C-H-C structures, the *n-*alkanes also fixate atmospheric hydrogen, whose isotopic ratios fluctuate as a measure of the amount and source of precipitation and atmospheric temperature and evapotranspiration.^70^^,^^71^^,^^72^^,^^73^ Thus, the δ^2^H (also referred to as δD) of specific *n*-alkanes may be used as a proxy for temperature and precipitation.^72^^,^^74^ However, the hydrogen stable isotope composition of *n*-alkanes can also be influenced by other factors such as plant type^72^ or the salinity in soil moisture and water bodies, which affect the fractionation processes that take place during the uptake of water and nutrients by plants.^75^ The stable isotopic signature of the carbon atoms in *n-*alkane chains is also informative, reflecting plant respiration pathways and the isotopic composition of atmospheric carbon. Thus, the *n-*alkane δ^13^C can be used as a proxy for vegetation mass and type, and water availability.^76^ In paleoenvironmental studies of archaeological sedimentary sequences, compound-specific stable isotope data are assessed on a relative scale where the focus is on fluctuations within a known time range, usually based on a set of sediment samples collected at small regular intervals across a stratigraphic column. Intervals may vary depending on the chronological framework and sampling constraints.

In the past decade, several groundbreaking case studies have presented paleoenvironmental interpretations of sedimentary lipid biomarker data, helping advance our understanding of particular archaeological contexts. One study focusing on *n*-alkanes and hopanoids examined Serbian Neolithic “Obrovac”-type settlements, typically situated on mounds surrounded by ditches and interpreted as either permanent wetland settlements or seasonal pastoralist or farming sites. The analysis identified macrophytes linked to floodplains and long-chain *n*-alkanes, possibly from grasses, indicative of terrestrial plants. This suggests a biodiverse landscape combining wetland and terrestrial vegetation during the periods of occupation.^77^ Other studies focus on *n*-alkane data derived from stratified Pleistocene archaeological sequences.^78^^,^^79^^,^^80^^,^^81^^,^^82^^,^^83^ In these studies, the presence of *n*-alkane biomarkers representative of plant types and their ecological settings offers clues about the vegetation and landscapes that prevailed during deposition of their associated sediment, which is in turn associated with the humans that lived there during that time. For example, at Diepkloof, a Middle Stone Age site in South Africa’s Western Cape, a change in the predominant leaf-wax *n*-alkane chain length and in leaf wax δD (δD_wax_) coincides with a segment of the stratigraphic sequence containing a specific lithic industry known as the Howiesons Poort, and suggests input of more arid-adapted vegetation types.^78^ In another study from the same region, Patalano and colleagues^84^ approached the paleohydrology and paleovegetation of a sedimentary sequence from the archaeological site of Ha Makotoko, a rock shelter site in Lesotho that spans the Late Pleistocene-Late Holocene. Their δ^13^C_wax_ data revealed a constant C_3_ vegetation input throughout the Pleistocene and Early Holocene and a change to C_4_ grassland vegetation only around 500 years ago, while δD_wax_ indicated cooler, drier conditions in the Pleistocene and Early Holocene compared to the Late Holocene. Different indices applied to the *n*-alkane record, i.e., average chain length (ACL) and carbon preference index (CPI), point to temperature as the controlling factor of vegetation distribution over this long period of time and suggest that South Africa was a scenario of local landscape changes.^84^ For a review on the importance of plant wax biomarker research in our understanding of Pleistocene climate, vegetation fluctuations and freshwater availability at a regional scale with important implications for human evolution, see Patalano et al. (2021).^85^

Recent geoarchaeological research has also seen exploration of other paleoenvironmental biomarkers commonly studied by climate scientists in non-archaeological sediments. These biomarkers include glycerol dialkyl glycerol tetraether lipids (GDGTs), which are compounds synthesized by bacteria in soils (branched GDGTs) or aquatic environments (isoprenoid GDGTs) that are sensitive to humidity, temperature, and pH variations.^86^ They accumulate in soils and sediments, and have been used to understand ancient terrestrial temperature and climate changes, to identify depositional environments and to distinguish between marine and terrestrial inputs. Their detection requires high performance liquid chromatography-mass spectrometry (HPLC-MS).^52^ In a recent study, Kielhofer and colleagues^87^ used branched GDGTs (brGDGTs) to explore temperature variations in central Alaska over the last 14,000 years. This region plays a key role in debates about the earliest human settlement of the Americas. Their analysis revealed a stable temperature record, suggesting that the population fluctuations evident in the archaeological record are not linked to climate change.^87^ Other informative compounds are specific steradienes, A-norsteranes, fern-8-ene, and chromans, which derive from aquatic organisms and as such, may be utilized as biomarkers.^88^ Their application to terrestrial archaeological sediments is incipient and promising.^82^

### Challenges in applying paleoenvironmental research to archaeological sedimentary contexts

**Figure 2:**
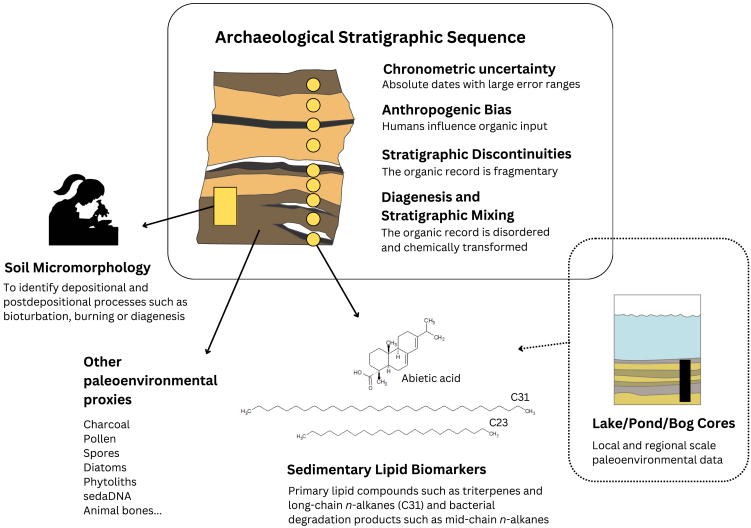
Infographic highlighting some of the complicating factors of paleoenvironmental lipid biomarker research applied to archaeological stratigraphic sequences First, absolute dates are often patchy and with large error ranges, which may result in a weak chronostratigraphic framework. Second, the organic matter contained in the sediment may be from a natural or an anthropogenic source, hindering direct paleoenvironmental interpretation. Third, archaeological stratigraphic sequences are typically discontinuous, hampering a continuous paleoenvironmental record, and finally, they encompass a great deal of postdepositional processes including chemical transformations due to diagenesis and physical reworking at different scales, which obliterates the primary lipid biomarker record. One way to confront these challenges is to conduct a multiproxy approach, integrating sedimentary lipid biomarker data with data from soil micromorphology, which provides information about depositional and postdepositional processes, and environmental proxies from other materials such as charcoal, pollen, phytoliths or macro and micro-fauna. Supporting paleoenvironmental proxies obtained from non-archaeological sediments such as lake, pond and bog deposits, which are more continuous, provide valuable complementary information.

Although the archaeological source and narrow time intervals (millennial-scale and less) of sedimentary lipid biomarker data obtained from stratified deposits provide a robust foundation for investigating human-climate interactions, this line of research entails several challenges (Figure 2). The first challenge entails the patchiness and large error ranges in absolute dates commonly associated with archaeological sequences. Often, it is difficult to anchor paleoenvironmental proxy variability in time, especially in deeply stratified Palaeolithic contexts. In such cases, turning to other paleonvironmental proxies may provide guidelines for interpretation. In a recent sedimentary lipid biomarker study aimed at paleoenvironmental reconstruction of the deposit from Crvena Stijena Cave, a 25 m-thick archaeological sequence spanning the upper Pleistocene and without fine-grained absolute chronometric data, an interpretation of the *n*-alkane isotopic variability as representative of the MIS 3 climatic period was supported by complementary micromorphological, anthracological, faunal, and total elemental organic data (TIC, TOC, and TS).^83^ In this case, the anthracological and faunal record were a particularly helpful relative chronostratigraphic guide to anchor the biomarker data.

A key challenge in using archaeological stratigraphic sequences for paleoenvironmental reconstruction lies in assessing anthropogenic bias and identifying subtle stratigraphic discontinuities. Archaeological sedimentary deposits often contain organic matter from mixed sources, making it difficult to separate inputs from natural environments and human activities. For example, the presence of biomarkers from a specific plant may reflect either the surrounding vegetation or plants introduced by humans, complicating interpretations. This situation has been highlighted by different researchers.^84^^,^^89^ Additionally, unlike lake sediments, which typically accumulate in relatively stable environments, archaeological deposits are exposed to terrestrial surface dynamics, such as erosion, sedimentary stasis, and bioturbation. These processes can create stratigraphic discontinuities or mix materials from different time periods, potentially leading to inaccurate reconstructions unless precise dating is available. This issue is not unique to biomarker analysis but also affects other methods, such as anthracology,^90^ or phytolith studies.^91^ Combining data from archaeological contexts with nearby lacustrine sediment cores, which often provide continuous or near-continuous paleoenvironmental records, can help address these challenges.^92^^,^^93^ Such an approach allows for robust reconstructions and can clarify potential anthropogenic biases in the archaeological record.

Additionally, in archaeological sedimentary deposits there is the risk of obtaining a misleading plant biomarker signal as an effect of diagenesis or stratigraphic reworking. Such postdepositional processes are hard to avoid due to the time-averaged, terrestrial, detrital, mixed nature of archaeological sediments, although they are also common in non-archaeological sediments. As a result, we encounter the challenge of assessing whether absence or a relative stratigraphic decrease in the concentration of a given lipid biomarker represents true absence of the biological source or its *in situ* degradation. Furthermore, *in situ* decay and diagenesis may affect isotope fractionation. One way to approach this issue is through identification of lipid compounds resulting from bacterial degradation^94^^,^^95^ (and see in the study by Patalano et al.,^85^ for a review of this issue). Another way to overcome the problem is to apply a microcontextual approach that integrates lipid biomarkers, other paleoenvironmental proxies, and soil micromorphology, a multidisciplinary technique that provides microstratigraphic information about depositional and postdepositional sedimentary processes^96^ and is widely used in geoarchaeology.^97^ The advantages of the multiproxy approach in paleoenvironmental interpretations has been was shown in a study of the archaeological sedimentary sequence from Abric del Pastor, a Middle Palaeolithic site in Spain, in which δ^13^C_wax_ and δD_wax_ data were coupled with anthracological, microvertebrate, macrofaunal, and soil micromorphological data.^80^ Micromorphological evidence from *in situ* anthropogenic fire in one of the stratigraphic layers was linked to the observed *n*-alkane degradation in that layer, the low occurrence of microscopic fresh rootlets ruled out modern bioturbation as a source of organic matter, the fresh state of the mineral microscopic fraction indicated relatively rapid sedimentation throughout the sequence, and the presence of weakly lenticular and granular microstructures indicative of cyclical freeze-thaw was congruent with δ^13^C_wax_ and δD_wax_ values suggestive of lower temperatures, as well as with the data from the other proxies. The results of this multiproxy microcontextual study are relevant to our understanding of the regional effect of the MIS 4 period of global cooling and its possible influence on Neanderthal disappearance. A different multiproxy study of Molleres II, a high-altitude site in the Eastern Pyrenees, revealed the existence of Neolithic animal enclosures, the persistence of pastoralist activity until the Middle Ages, and a present-day landscape strongly influenced by its anthropogenic history.^98^

Additional methodological challenges are posed by instrumental detection limits. For example, certain archaeological sediment samples with a low total carbon content may yield insufficient amounts of biomarkers for measuring isotopic ratios, particularly for those in smaller proportions within the sample. Despite the existing diversity of systems for measuring isotopes in fields such as chemistry, geology, environmental sciences, or biosciences,^99^ such as laser ablation-inductively coupled plasma-mass spectrometry, isotopic ratio infrared spectroscopy or isotopic ratio mass spectrometry (IRMS), to name a few, not all instruments can be used for any purpose. Therefore, this limitation can be addressed by enhancing instrumental sensitivity. In recent decades, isotope geochemistry has undergone significant evolution with the introduction of multi-collector plasma mass spectrometry, enhancements in gas source mass spectrometry, advancements in infrared absorption spectroscopy, progress in nuclear magnetic resonance, and the utilization of high-precision ion microprobe techniques.^99^ These technological advancements encompass innovations such as multi-collector plasma source mass spectrometers (MS), absorption spectroscopy, the emergence of high-resolution multi-collector gas source MS, and natural abundance NMR techniques. These developments have facilitated the analysis of “nontraditional” stable isotopes (e.g., Mg, Fe, Cu^100^^,^^101^^,^^102^), the expansion of mass-independent isotope geochemistry, the establishment of clumped isotope geochemistry, and advancements in measuring position-specific isotope effects in organic compounds. Given the considerable potential of the new techniques, it is anticipated that they will become increasingly common in sedimentary analysis.

## Sedimentary lipids as windows to past human behavior: Diet, fire technology and settlement patterns

### Approaching plant and animal taxonomy through lipid biomarker compounds and compound-specific isotope ratios

**Figure 3:**
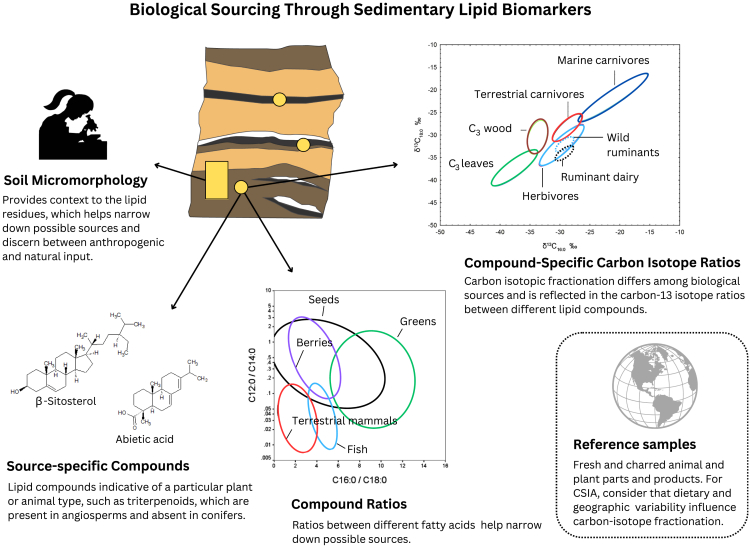
Infographic showing different ways to identify biological sources using lipid biomarkers Source-specific compounds: certain lipid compounds are specific to particular kinds of plants or animals. Compound ratios: ratios between different lipid compounds common in most plant and animal matter, such as fatty acids, are biomarkers of particular kinds of plant and animals (diagram modified from Eerkens, 2005^103^). Compound-specific carbon isotope ratios: the ratios between the carbon-13 isotopes of different common lipid compounds such as fatty acids, are also biomarkers of particular kinds of plants and animals and their products (diagram modified from different sources^8^^,^^15^^,^^16^^,^^18^^,^^19^^,^^105^^,^^106^^,^^107^). Due to the complex, mixed nature of archaeological sediment, it is advisable to incorporate soil micromorphology to provide context to the lipid residues and help narrow down possible sources. Biological sourcing relies on reference lipid biomarker data (compounds, compound ratios, and carbon-13 isotope ratios) from plant and animals and their products, not only fresh but also charred, as organic residues in archaeological sediment are often charred. For compound-specific isotope ratios, it is important to have reference data from different geographic regions, as latitude and altitude are two important environmental variables influencing carbon isotope fractionation. Likewise, animal diet in different ecosystems should also be considered because plant communities are influenced by precipitation and temperature, which strongly determine the carbon isotopic signature.^108^

Identifying the taxonomic composition of plants and animals in archaeological contexts is crucial for reconstructing past environments and interpreting various aspects of social and economic life, including the diets of humans and domestic animals, their settlement and mobility patterns, fuel sources, and other technological elements. Though now advancing rapidly with the development of paleogenetics and paleoproteomics, this area of analysis began with lipid residue analysis.^6^^,^^7^ By identifying and quantifying specific lipid compounds and examining the ratios between them—such as *n*-alkanes or fatty acids^6^^,^^103^—or between compound-specific carbon isotopes^8^^,^^15^^,^^16^^,^^18^^,^^19^^,^^104^^,^^105^^,^^106^ (Figure 3), researchers have accessed new insights into ancient diets and resource use. The current literature shows a growing number of multiproxy studies that incorporate microstratigraphic geoarchaeological techniques, and reveals that applying biomarker analysis in archaeological sediments requires a coupled microstratigraphic approach to inform on site formation processes. To interpret past human behavior accurately, it is essential to determine whether animal or plant residues identified through lipid analysis are anthropogenic and whether they are *in situ*.

Exploring lipid biomarkers in the archaeological sedimentary record to identify biological sources is a challenging task, considering the mixed, time-averaged nature of archaeological sediments. Studies using the presence of sedimentary lipid biomarker compounds in *non-archaeological* settings as proxies of human behavior or human impact on the landscape typically rely on a multiproxy approach and obtain their data from sediment cores, which can be analyzed at very small stratigraphic intervals.^109^^,^^110^^,^^111^ Interpreting the presence of individual lipid biomarker compounds in *archaeological* sediments requires microstratigraphic monitoring not only to approach the source of the lipid residue but also to discern between natural or anthropogenic input. The existing literature presents various examples of multiproxy, microstratigraphic studies aimed at understanding human behavior from diverse perspectives. For instance, a long-term analysis was conducted on different trenches at and near an Inuit site in Nunavik, Canada, to evaluate human impact on the Arctic landscape. This study identified sedimentary fatty acid enrichment associated with animal butchery practices dating back to the 19th century, linking these practices to local-scale soil transformation.^112^ Similarly, in a study focusing on Neanderthal behavior, researchers explored the complexity of Neanderthal hearths by analyzing the presence of lipid biomarker compounds within their specific anthropogenic context. This investigation revealed the presence of triterpenoids, angiosperm plant biomarkers, in the black layers of Neanderthal hearths, which were identified, through other proxies, as fueled with pine wood.^90^ This discovery underscores the distinction between the burning event and the underlying soil surface.

Different attempts have been made to discriminate types or classes of plants and animals from the *ratios* between different lipid compounds in sediment samples, particularly between hexadecanoic acid (palmitic acid, C_16:0_) and octadecanoic acid (stearic acid, C_18:0_), which are naturally abundant fatty acids in living organisms, including bacteria, fungi, plants, and animals. March and colleagues have used this method to explore dietary sources in archaeological sediments from hearths of different time periods and regions, discerning between plants and animals and interpreting the input of different animals.^113^^,^^114^^,^^115^^,^^116^^,^^117^ In a study of two Iron and Bronze Age sites in Thailand, researchers identified domestic occupation surfaces based on the presence of different fatty acids, particularly odd-chain fatty acids (C15:0 and C17:0) in ratios suggesting a ruminant animal source.^118^ These ratios are often used to discern between terrestrial vs. marine plants and animals, or among different types or classes of plants and animals because they reflect specific metabolic pathways of atmospheric carbon absorption and fractionation either directly (plants) or through the food chain (animals).^6^^,^^104^^,^^119^ Nonetheless, these compounds are prone to alteration and degradation, posing challenges in accurately attributing residues to their respective original food sources.^103^^,^^120^^,^^121^^,^^122^

Besides lipid compound characterization, GC-MS combined with CSIA, particularly δ^13^C, is commonly applied to archaeological sediment samples to discern among animal and plant sources or determine plant or animal types in archaeological sediments from different regions and time periods. One way to approach the biological source is through the δ^13^C isotopic signal of particular long-chain *n*-alkanes or of different fatty acids. Biological isotopic fractionation and hence compound-specific isotopic ratios are sufficiently resistant to microbial and aerobic degradation and diagenesis to have preservation potential in archaeological contexts. However, the preservation potential differs among lipid compound types. For example, plant sterols in soils have shown to have a better preservation potential than *n*-alkyl molecules.^123^ In a study of Cape Espenberg, an early prehistoric beach dune site in northwest Alaska with poor bone preservation, samples of the archaeological dune sands yielded fatty acid residues with C_16:0_ to C_18:0_ δ^13^C values in the range of marine animal fats, advancing our knowledge on the diet and hunting strategies of prehistoric societies in Arctic America.^124^ Altogether, the published compound-specific isotope biomarker research in archaeological contexts deals mostly with animal sources and there is a scarcity of work focusing on plants.^107^^,^^125^

### Fecal biomarkers in archaeological sediments

**Figure 4:**
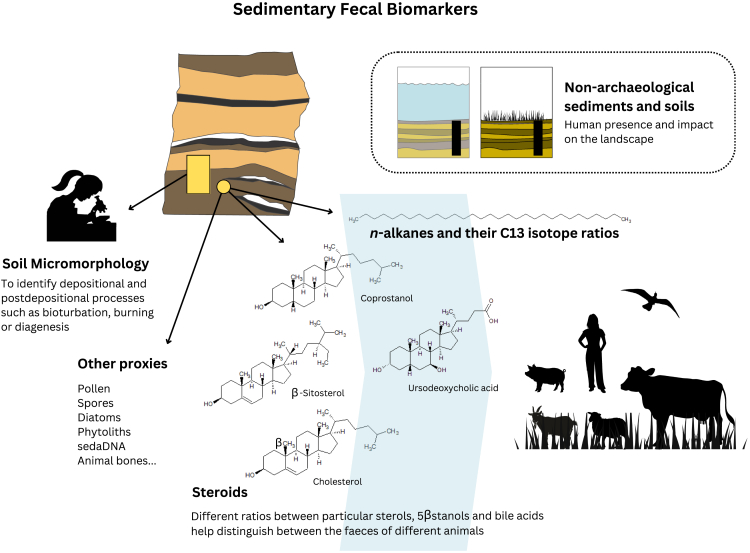
Infographic illustrating how archaeological fecal lipid biomarkers can help discern among different sources of animal feces and animal diets The current methods rely on ratios between different steroids, including sterols, 5β-stanols, stanones and bile acids. Incorporating *n*-alkanes and their δ^13^C isotope values provide further dietary information. Microscopic particles and micromorphological analysis offer context and further information on animals and diets. Sampling and applying the same analytical suite to non-archaeological soils and sediments contributes information about human presence and impact on the landscape.

Excrements are readily broken down and incorporated into archaeological sediments as microscopic fragments or molecular residues, and their source can be determined through sedimentary fecal lipid biomarker analysis. The common presence of excremental residues in archaeological sediments has led to a focus on archaeological fecal biomarkers, a rapidly growing area of study that yields important information about plant and animal taxonomy in order to clarify past human and animal diets and husbandry practices. It is possible to approach an identification of the source animal (carnivore vs. herbivore) and in the case of herbivores, the dietary plant source. The relevant biomarkers may be present in different lipid fractions. Steroids, which are among the most resistant lipid compounds, are commonly used to establish excremental sources of coprolite and sediment samples.^113^^,^^114^^,^^126^^,^^127^^,^^128^^,^^129^ The method is based on a series of ratios between different sterols, 5β-stanols (fecal derivatives of sterols; specifically, coprostanol and 5β-stigmastanol), and bile acids (Figure 4). These ratios assess the presence of fecal matter^5^^,^^127^ and ruminants,^126^^,^^127^ and help discriminate among different animal sources including herbivore vs. pig vs. human^127^^,^^130^ or ruminant vs. pig or human.^131^ 5β-stanols are also naturally present in soils and sediments in minor proportions from the reduction of Δ^5^-sterols, a fact that must be considered in fecal biomarker exploration.^132^ The method has been widely applied to archaeological coprolite samples, contributing to major debates such as the earliest peopling of the Americas.^133^^,^^134^ See Shillito et al., 2020^135^ for a comprehensive review on multianalytical archaeological coprolite analysis.

Preservation of fecal lipid biomarkers in non-archaeological lacustrine and terrestrial sedimentary deposits has been previously shown by research aimed at assessing fecal contamination from humans and domesticated animals in water, sediments, and soils^136^^,^^137^^,^^138^^,^^139^ or seeking to detect human presence and impact on the landscape.^98^^,^^110^^,^^140^^,^^141^^,^^142^^,^^143^^,^^144^^,^^145^^,^^146^^,^^147^^,^^148^ The potential of fecal biomarker analysis in archaeological sediments was set forth by Evershed and colleagues^5^^,^^149^ and since then, the high preservation potential of sedimentary steroids has been demonstrated by different case studies. One study reports stanols preserved in Middle Palaeolithic sediments, interpreted as representative of Neanderthal excrements.^150^ This study is microcontextual, linking the biomarker data with microstratigraphic evidence of microscopic coprolites on a human occupation surface. Additional microcontextual investigations have been conducted in other sites, such as the Iron Age wetland settlement of Black Loch of Myrton, in Scotland, where combined steroid biomarkers, archaeoentomology and micromorphology from selected floor deposits informed of the functionality of the houses as cattle stables during certain periods of the settlement’s history.^151^ Other microcontextual multiproxy studies of indigenous archaeological sites in the Canary Islands have identified the presence of sheep dung in stabling deposits and house floors.^152^^,^^153^ In these cases, although fecal biomarker ratios do not discriminate between sheep or cow, the micromorphological differences between both are distinct, and cattle was first introduced in the Canary Islands by Europeans, thus its presence in indigenous sites can be ruled out.

The microcontextual approach is particularly helpful in the investigation of fecal biomarkers in sediments due to the current debates around the reliability of sterols as fecal biomarkers. A recent multiproxy investigation of fecal biomarkers coupling *n*-alkanes, 5β-stanols and bile acids highlighted the importance of bile acids as fecal biomarkers given the confounding representation of 5β-stanols, which incorporate non-fecal sources, especially in older deposits.^147^ Furthermore, the organisms’ diets have an influence on the resulting sterol profile.^154^ Crucially, diet composition can vary across regions and seasons, and is seldom known in archaeological contexts. For an additional review of methods and a discussion around animal dietary and fecal lipid biomarkers, see Vázquez et al., 2021.^155^ Another set of multiproxy studies involving Early Medieval lake settlements in Ireland (Celtic crannogs) report paleoenvironmental and behavioral proxies obtained through multi-technique paleolimnological and biomolecular analysis, integrating fecal lipid biomarkers with sedimentary ancient DNA (sedaDNA), mineralogical data and microparticles (spores, diatoms and microcharcoal and others), contributing valuable information on animal husbandry and food resource exploitation.^156^ This study is also an example of the great potential of multiproxy geoarchaeological research as a strategy to obtain data on past human behavior and environments in a quick, minimally destructive way at sites at which archaeological excavation is not possible or that risk destruction.

Looking at other lipid fractions, reference studies in contemporary and ethnoarchaeological contexts associated with herbivore excrements, such as animal pens or buried soils representing excrement-rich prairies or forests have shown that the *n*-alkane composition and *n*-alkane δ^13^C values are informative of the group or family of plants consumed by the animals.^157^^,^^158^^,^^159^^,^^160^^,^^161^ This work is the foundation for pioneering case studies of archaeological sheep and goat pens from different regions and time periods that are starting to shed some light on the herds’ diets and local vegetation, contributing to our archaeological knowledge of pastoralist societies.^98^^,^^162^^,^^163^

### Pyrogenic lipid biomarkers or “pyromarkers” in archaeological sediments

**Figure 5:**
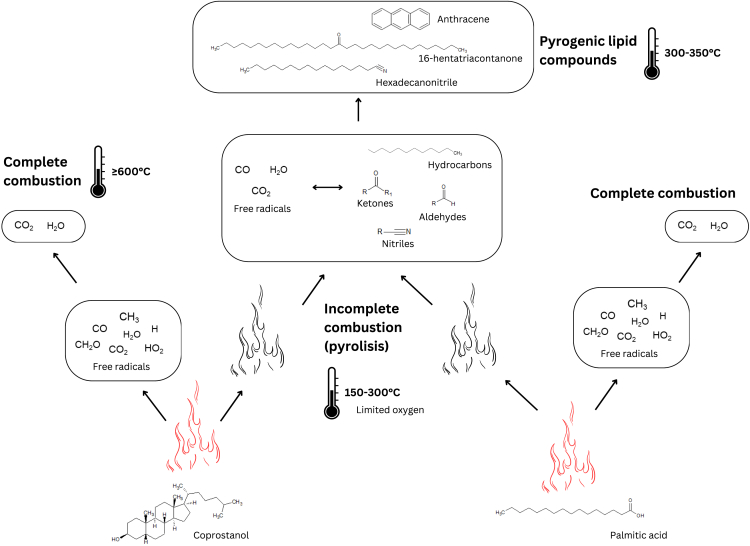
Simplified diagram illustrating the primary chemical transformations involved in lipid compound combustion in oxygen-rich environments such as in a flaming fire Heat exceeding approximately 150°C, causes the cleavage of weak bonds, resulting in the formation and volatilization of free radicals and more complex molecules derived from the original compound’s core structure. In well-oxygenated conditions with temperatures reaching 600°C or higher, this process may initiate a chain reaction, leading to complete combustion of the entire chemical structure and the release of water and carbon dioxide. Conversely, under poorly oxygenated conditions or at temperatures between 300°C and 350°C, pyrolysis or charring occurs. In this case, there is less volatilization and free radicals may combine with each other and with other complex molecules to form pyrogenic lipid compounds, predominantly aromatic compounds. Our current knowledge of the archaeological preservation of pyrogenic lipid compounds remains limited.

**Figure 6:**
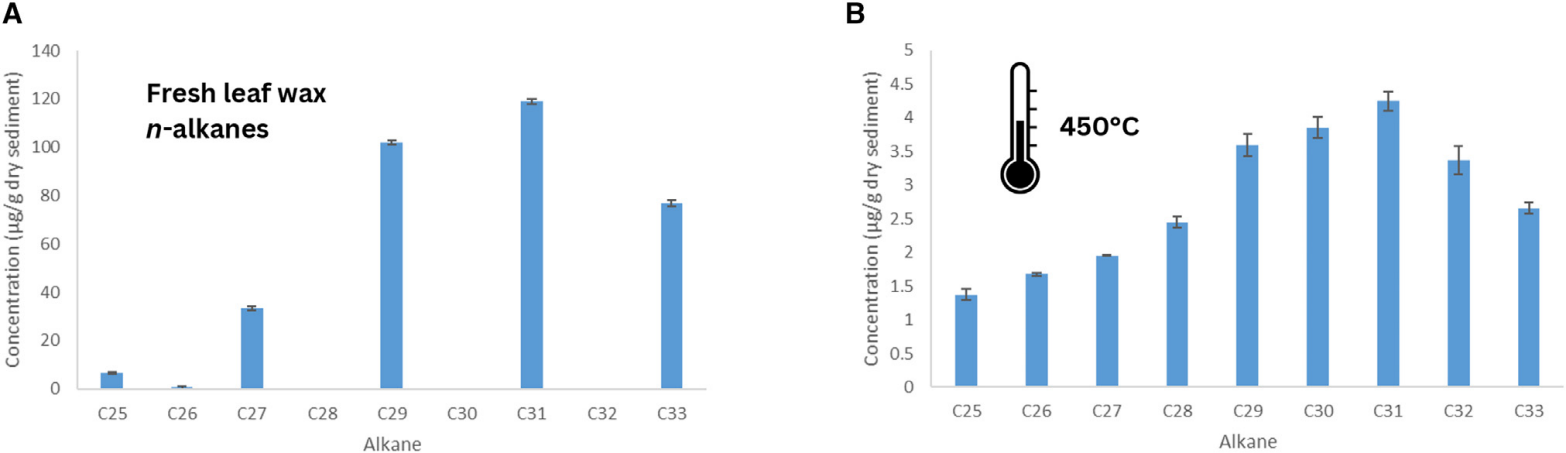
Hitograms of n-alkane lipid profiles showing a comparison between unburnt and burnt samples Histograms of experimenta*l n*-alkane profiles from: (A) fresh leaf waxes, and (B) leaf waxes burnt at 450°C under partially oxygenated conditions. The error bars correspond to the uncertainties obtained when calculating the concentrations using calibration curves.

Humans have controlled and relied on fire since the Pleistocene, and combustion residues are found in archaeological sediments across nearly all regions and time periods. These residues, which indicate the presence and technology of fire use, contain an organic fraction with significant potential for lipid biomarker analysis. Certain biomarkers, may be identifiable despite low-temperature alteration, while biomass burning at higher temperatures can lead to the formation of pyrogenic lipid biomarkers^164^^,^^165^^,^^166^^,^^167^^,^^168^^,^^169^^,^^170^^,^^171^^,^^172^^,^^173^ (Figure 5). For example, conifer burning produces retene, a polycyclic aromatic hydrocarbon (PAH), and dehydroabietic acid through the oxidation of abietic acid, the natural diterpenoid lipid compound in pine resin.^174^^,^^175^^,^^176^ Grass burning results in identifiable changes in the *n*-alkane lipid profile. Long carbon chains are broken into shorter ones, and the characteristic predominance of odd-numbered long carbon chains is replaced by a balanced or “smoothed” odd/even distribution pattern^171^ (Figure 6). In a synthesis of a long-term experimental program involving lipid biomarker investigations and anthropogenic fire, March et al. (2014)^172^ report changes in different lipid fractions, notably the mentioned changes in *n*-alkane distribution and a decrease in the ratio of C_16:0_ to C_18:0_. For a comprehensive review on the lipid compounds produced in biomass pyrolysis and their sources, see Simoneit (2002).^166^

The compounds of soot and char, which result from combustion at relatively low temperatures (<300°C–350°C) or under poor oxygenation (pyrolysis), show great potential as targets of archaeological research because they are shielded from oxidation and microbial degradation when compared to fresh organic matter.^177^ Given the evidence of human-controlled fire since the Palaeolithic, we can generally expect thermal degradation in a significant proportion of the lipid molecular and isotopic record of archaeological sediments. Not surprisingly, there is a considerable amount of published sedimentary lipid biomarker research focusing on the experimental or archaeological study of sediment samples from hearths or combustion structures. These investigations hold great potential to contribute clues about ancient pyrotechnology and also about ancient diets, due to the direct link between fire and food. Hearths have been foci of food discard and cooking since Paleolithic times. In recent years, there has been a considerable amount of lipid biomarker research exploring the effect of fire on lipid compounds, the formation of fire-derived compounds, and their presence in archaeological sediments, particularly organic-rich black sediments derived from low-temperature burning. Pioneering research showed the preservation potential of different lipid compounds in archaeological sediments from hearths. Some studies reported the presence of odd-over-even *n*-alkane profiles and identifiable fatty acids and sterols in sediment samples from Paleolithic hearths^114^^,^^115^^,^^117^^,^^172^^,^^178^), which allowed them to approach Paleolithic environments and diets through biomarker analysis. These studies showed that the saturated fatty acid content was sufficiently well preserved and in amounts that allowed compound-specific isotopic ratio analysis.

Experiments involving the analysis of sediments from hearths to characterize the lipid traces of different cooking techniques and fuel sources have reported variability in lipid compounds, including pyrogenic compounds.^117^^,^^172^^,^^179^^,^^180^^,^^181^ For example, in an experimental study to explore differences between wood and bone fueled fires, Léjay et al. (2016)^181^ sampled the fires’ sediment substrate and reported the presence of aromatic and phenolic compounds only in wood fueled fires, while bone fueled fires yielded a series of pyrogenic lipid compounds derived from fatty acid breakdown. More recent experimental work is partly grounded on the apparent high potential of the black layers from archaeological combustion features to preserve organic matter due to their carbonaceous nature, the relatively low burning temperatures they generally represent (<400°C), and their close association with human occupation surfaces.^182^ These investigations corroborate the potential of some of the previously reported lipid biomarkers. One study corroborated the presence of saturated long-chain ketones in sediments from bone fueled hearths, their formation at temperatures above 300°C, and the preservation of the molecules’ diagnostic δ^13^C values, strengthening their potential as pyrogenic lipid biomarkers (lipid pyromarkers) of bone fuel.^183^ This discovery holds particular significance due to its relevance in archaeological settings found in Arctic regions or Pleistocene glacial environments where wood is scarce or absent, and bone fuel is probable. Other research has focused on *n*-alkanes, reporting their anatomical part-dependent differential thermal degradation in terrestrial plants, where leaf wax *n*-alkanes resisted higher temperatures than woody xylem *n*-alkanes.^184^ Another study showed the temperature ranges at which δ^2^H *n*-alkane values change due to thermal degradation, proposing a 300°C threshold.^185^

Adding to the evidence showing the good preservation potential of sedimentary lipid compounds and their isotopic signatures at 300°C–350°C, other experiments report δ^13^C values of fatty acids from plant burning and their potential to distinguish between animal and plant sources and between wood and leaves.^107^ The same study also reported the formation of *n*-alkyl nitriles and *n*-alkyl amides at 350°C during the combustion of different plant anatomical parts, and their presence in Palaeolithic fire-derived black sediments.^107^ The presence of nitriles at specific temperature ranges and their preservation in ancient sediments raise their analytical potential as archaeological pyromarkers resulting from both plant and animal burning because these compounds also form from a reaction between amino acids and carboxilic acid in combustion contexts involving meat.^186^ The current studies suggest that the black layers of archaeological combustion structures are a good place to sample to explore such pyromarkers, and the challenge lies in distinguishing between plant and animal sources because as previously mentioned, most of the current literature on CSIA focuses on discerning among different animal sources.^8^^,^^116^

Recent works in archaeological settings from Palaeolithic to historic times incorporate microcontextual information^89^^,^^90^^,^^163^^,^^187^^,^^188^^,^^189^ and provide interpretations of fuel sources, plant sources of charred soil cover, and burning temperatures based on the presence of specific compounds and their δ^13^C values. For example, Tomé et al. (2022)^89^ built a lipid biomarker reference collection of endemic high-altitude (2290 m asl) plants in Tenerife, Canary Islands, which allowed them to identify the use of *Juniperus turbinata* or sabina wood as a source of fuel based on its characteristic δ^13^C C_16:0_ and C_18:0_ values. These fuel residues were found in successive layers of trampled ashy sediment that included domestic refuse, hearth residues and sheep or goat dung, all of which speaks for the domestic life of pastoralists groups that occupied highland lava tube shelters before the arrival of Europeans in the 15^th^ century.

Polycyclic aromatic hydrocarbons (PAHs), volatile pyrogenic molecules derived from the combustion of organic matter and found in different substrates including coal, bitumen, soot, and smoke, have also gained significant relevance in recent geoarchaeological studies. These compounds are highly lipophilic and have a low solubility in water. This is the reason why they can be extracted together with lipid biomarkers. They are commonly found in smoke from wild and anthropogenic fires and have been investigated by environmental and fire scientists (see a review by Lima et al.^190^). This research has produced experimental data on PAHs from different wood sources^191^^,^^192^^,^^193^^,^^194^ and bitumen used in anthropogenic contexts.^195^ PAHs in sediments have been documented in non-archaeological lacustrine and terrestrial sequences, where they record the occurrence of wild and anthropogenic fire.^111^^,^^141^^,^^143^^,^^196^^,^^197^^,^^198^^,^^199^^,^^200^ Some of these studies showed that particular PAHs are informative of distance to the combustion source or area burned. For example, Halsall et al.^196^ modeled the atmospheric transport of fluorene, phenanthrene, fluoranthene, and benzo[*a*]pyrene over a 5-day period from the UK to the Russian Arctic and found that only fluorene and phenanthrene, which are lighter, would reach the Arctic. Another study assessed the efficacy of different PAHs as proxies to estimate the areas burned by paleofires, reporting that naphthalene, acenaphthene, anthracene, and fluorene are local proxies of area burned (within 40 km), while phenanthrene, chrysene and benzo[*g,h,i*]perylene cover areas up to 75 km and benzo[*b*]fluoranthene and benzo[*k*]fluoranthene cover areas up to 150 km^200^. A study examining sediment samples from the Middle Palaeolithic site of Lusakert 1, Armenia, researchers observed contrasting distributions of low molecular weight PAHs (lPAHs) associated with wildfires and high molecular weight PAHs (hPAHs) indicative of anthropogenic hearths. The findings revealed that hPAHs were more prevalent during periods of intense human occupation at the site, while lPAHs were less common. The researchers interpreted these results as suggesting that the human groups inhabiting the area did not rely on wildfires.^79^ Another recent study identified alkylated 3-ring PAHs and norabietane derivatives indicative of conifer biomass burning in sediment samples from two hearth-like features at Valdocarros II, an Acheulean site in Spain dated to around 245 kya.^201^ These data contribute to our knowledge of human-made fire and fire technology in the course of human evolution. There are additional fire proxies with potential for archaeological research that remain underexplored (see Simoneit, 2002^166^ for a comprehensive review). One such compound is levoglucosan, a monosaccharide anhydride produced in cellulose burning.^202^^,^^203^ Although it is not a lipid, sedimentary levoglucosan can be extracted and analyzed using chromatographic techniques.^204^ Also, recent experimental, ethnographic, and archaeological studies have identified previously undocumented pyromarkers in pottery, such as oxo fatty acids and ω-(2-alkylcyclopentyl) alkanoic acids, which could be explored further in sedimentary contexts.^205^^,^^206^

This overview of the recent literature on archaeological sedimentary lipid biomarkers and combustion reveals a growing field with different promising avenues of research. One is the basic research into the thermal degradation of lipid biomarkers. Distinguishing between the thermal degradation of plant and animal sources and their aerobic or diagenetic degradation is crucial to behavioral and paleoenvironmental interpretations. Does a degraded lipid compound represent aerobic, diagenetic or thermal degradation? In some cases, there are methods to resolve such issues. For example, identification of degraded *n*-alkanes or alcohols or fatty acids in association with pyrogenic aromatic compounds might corroborate thermal degradation. Unfortunately, pyrogenic aromatic compounds are generally scarce due to their high volatility, so their absence does not rule out thermal degradation.

## Methodological aspects of archaeological sediment lipid analysis

Recent published experiments demonstrate a focus on refining and tuning existing methodologies to suit the unique characteristics of archaeological sedimentary contexts, which are substantially more open, mixed, and dynamic substrates than archaeological objects such as pottery or stone. Method optimization in archaeological sedimentary lipid analysis faces several challenges, including enhanced sample collection, lipid extraction and separation, analyte injection, detection, measurement, and quantification. In this section, we provide guidelines for novice analysts on the different steps involved in lipid biomarker analysis of archaeological sediments based on a review of relevant literature. Table 1 presents a selection of key analytical parameters for different sedimentary lipid biomarkers and pyromarkers.

**Table 1:** Selected key analytical parameters for different sedimentary lipid biomarkers and pyromarkers

Analytes	Extraction protocol	Determination method	ISs	Reference
PAHs, monosaccaride anhydrides, plant sterols, fecal sterols and stanols	ASE (diatomaceous earth, 150°C and 1500 psi, DCM or DCM: MeOH 9:1 v/v, two times) SPE (silica gel, 2 g)	GC-MS (derivatization with BSTFA + TMCS 99:1 v/v for alcohols) IC-MS	^13^C_6_-Cholesterol,^13^C_6_-Acenaphtylene,^13^C_6_-Phenanthrene,^13^C_4_-Benzo(*a*)pyrene,^13^C_6_-Levoglucosan	Argiriadis et al., 2018
Sterols and stanols	Soxhlet (acetone:n-hexane 1:1 v/v for 20 h)	GC-MS (derivatization with MSTFA + TMSI)	2,2,4,4-d_4_-5β-cholestanol	Benfenati et al., 1994
Fatty acids	SLE (chloroform:MeOH:H_2_O 4:2:1 v/v/v)	GC-MS (derivatization with MeOH:H_2_SO_4_ 99:1 v/v)	Nonadecanoic acid	Bernier et al., 2017
Sterols, stanols, stanones and bile acids	Soxhlet (150 mL DCM:MeOH 2:1 v/v for 36 h) Saponification (3.5 mL 0.7 M KOH in MeOH 10–14 h at room temperature) LLE (3x15 mL chloroform, acidification (pH ≤ 2), 3x15 mL chloroform) Methylation (1 mL dry 1.25 M HCl in MeOH 80°C for 2 h -acidic fraction-) SPE (silica gel)	GC-MS (derivatization with BSTFA + TSIM)	5α-pregnan-3β-ol, 5α-pregnan-3-one, 7α,12α-dihydroxy-5β-cholanoic acid, 5α-cholestane	Birk et al., 2012
*n*-Alkanes, stanols, Δ5-sterols and bile acids	Soxhlet (150 mL DCM:MeOH 2:1 v/v for 36 h) Saponification (3.5 mL 0.7 M KOH in MeOH 10–14 h at room temperature) LLE (3x15 mL chloroform, acidification (pH ≤ 2), 3x15 mL chloroform) Methylation (1 mL dry 1.25 M HCl in MeOH 80°C for 2 h -acidic fraction-) SPE (silica gel)	GC-MS (derivatization with BSTFA + TSIM)	Hexatriacontane, pregnanol, isodeoxycholic acid and cholestane	Birk et al., 2021
*n*-Alkanes and PAHs	Soxhlet (300 mL DCM:MeOH 2:1 v/v for 48 h) Saponification (5 mL of 1 M KOH in MeOH at 85°C for 2 h) LLE SPE (silica gel, 1.25 g)	GC-FID and GC-MS GC-IRMS	–	Brittingham et al., 2019
Bile acids	MAE (DCM:MeOH, 2:1, v/v) Saponification (5 M NaOH in MeOH) SPE (aminopropyl) Methylation (TMSDAM in toluene/MeOH 4:1 v/v) SPE (silica gel)	GC-FID and GC-MS (derivatization with BSTFA + TMCS 99:1 v/v)	androstanol and hyocholic acid	Brown et al., 2021
Carboxylic acids (fatty acids, isoprenoid fatty acids, ω-(*o*-alkylphenyl)alkanoic acids, α,ω-dicarboxylic acids)	UAE (15 mL chloroform:MeOH:H_2_O 1:2:0.8 v/v/v, 2 × 15 min) LLE (MeOH:H_2_O 1:1 v/v) Methylation (5 mL 1.25 M HCl in MeOH) LLE (3x 3 mL hexane)	GC-MS GC-IRMS	–	Buonasera et al., 2015
Sterols and bile acids	Soxhlet (150 mL DCM:MeOH 2:1 v/v for 24 h) Saponification (5 mL of 5 M KOH in 90% MeOH, 1h) Acidification (pH 3–4) LLE (3x10 mL chloroform) SPE (aminopropyl, 500 mg) Bile acids: Methylation (10 mL diazomethane in ether, room temperature overnight), SPE (silica gel, 600 mg) Sterols: SPE (silica gel)	GC-MS and GC-FID (derivatization pyridine:HMDS:TMCS 9:3:1 (v/v/v)	Hyocholic acid and 5β-pregnanol	Elhmmali et al., 2000
Glycerol dialkyl glycerol tetraethers	UAE (3 x MeOH, 3 x DCM:MeOH 1:1 v/v, 3 x DCM) SPE (alumina)	HPLC-MS	–	Hopmans et al., 2004
Fatty acids	Acidic SLE (HCl 2 M in MeOH at 65°C 16 h) SLE (diethyl ether)	GC-MS	–	Kedrowski et al., 2009
*n*-Alkanes, branched alkanes, alkylresorcinols and alkyldiols	ASE (DCM:MeOH 9:1 v/v, 3 × 5 min at 100°C and 1500 psi) SPE (silica gel)	GC-MS (derivatization BSTFA) GC-IRMS	–	Magill et al., 2016
Alkenones (ketones)	Soxhlet (DCM:MeOH 3:1 v/v) ASE (DCM:MeOH 9:1 v/v, 1000 psi, 100°C for 25 min)	AMS	–	Ohkouchi et al., 2005
Ketones, *n*-alkyl nitriles, *n*-alkyl aldehydes, fatty acid methyl esters and free fatty acids	UAE (3x10 mL DCM:MeOH 9:1 v/v, 30 min) SPE (silica gel, 1g)	GC-MS GC-IRMS	5α-androstane and methyl nonadecanoate	Jambrina-Enqríquez et al., 2019
n-Alkanes, alcohols and fatty acids	UAE (3x10 mL DCM:MeOH 9:1 v/v, 30 min) SPE (silica gel, 1g) Methylation of fatty acids (5 mL MeOH +400 μL H_2_SO_4_)	GC-MS (derivatization BSTFA + TMCS 99:1 v/v + pyridine) GC-IRMS	5α-androstane, 5α-androstan-3-ol and methyl nonadecanoate	Leierer et al., 2019
Glycerol dialkyl glycerol tetraethers	ASE (DCM:MeOH 9:1 v/v, 3 × 10 min 100°C) SPE (silica gel)	UHPLC-MS	C_46_ GDGT	Liu et al., 2020
*n*-Alkanes, sterols and stanols, monoalkyl glycerol monoethers, lineal alcohols, fatty acids	ASE (DCM:MeOH 9:1 v/v, 3 × 15 min at 100°C and 1500 psi) Acid methanolysis (0.5 M HCl in MeOH) LLE (hexane:DCM 4:1v/v) SPE	GC-MS (derivatization BSTFA) GC-IRMS	1-pentadecanol, *ai*-C_22_, epiandrosterone, 1-nonadecanol, and 2-methyloctadecanoic acid methyl ester	Sistiaga et al., 2020
Hormones and bile acids	MAE (25 mL DCM:MeOH 2:1 v/v, 1600 W) Saponification (5 mL of 2 M KOH (MeOH:H_2_O, 10:1 v/v, 100°C, 1.5 h) Acidification (2 ≤ pH ≤ 4) LLE (2 x 10 mL DCM)	HPLC-MS	^2^H_3_-estradiol and ^2^H_5_-lithocolic acid	Vallejo et al., 2022a

AMS, accelerator mass spectrometry; ASE, accelerated solvent extraction; BSTFA, N,O- bis(trimethylsilyl)trifluoroacetamide; DCM, dichloromethane, FID, flame ionization detector; GC, gas chromatography; HPLC, high-performance liquid chromatography; IC, ion chromatography; IRMS, isotope ratio mass spectrometry; LLE, liquid-liquid extraction; MAE, microwave assisted extraction; MS, mass spectrometry; MSTFA, N-methyl-N-trimethylsilyltrifluoroacetamide; PAH, polycyclic aromatic hydrocarbon; SLE, solid-liquid extraction; TMCS, trimethylchlorosilane; TMSDAM, trimethylsilyldiazomethane; TMSI, iodo-trimethylsilane; TSIM, N-trimethylsilylimidazole; UAE, ultrasound assisted extraction; UHPLC, ultra-high performance liquid chromatography.

### Fieldwork and sample collection

The initial challenge lies in the field: excavation profiles and surfaces should be cleaned before sampling by removing approximately 3 cm of sediment to avoid potential microbial contamination, as recommended for other bioarchaeological applications, such as ancient DNA studies.^83^^,^^207^^,^^208^ To mitigate the risk of contamination by phthalates and contemporary lipids, sediment samples for lipid analysis should be collected using sterilized metal tools and nitrile gloves.^83^ These samples should be placed in aluminum foil, Teflon, or glass vials and stored at 4°C or less to avoid bacterial degradation, something that has been reported by Brittingham et al.^209^ The required volume of sediment to obtain a detectable lipid content varies and is challenging to estimate. Typically, mixed sediments from archaeological sites that represent potential living surfaces or features such as black layers in combustion structures or other brown-black-colored sediments contain more than 0.5% total organic carbon (TOC). In such cases (and depending on target molecules), 5 g of sediment, approximately a spoonful, has proven to be adequate for lipid analysis (for example Leierer et al.,^90^^,^^187^). However, the white layers observed in combustion features, which typically constitute ash formed at temperatures exceeding 600°C, have been found to be devoid of lipids.^89^^,^^90^^,^^107^^,^^163^^,^^187^ In a specific case study focusing on heavily inorganic, almost white colored, calcareous archaeological sediments from a Paleolithic rock shelter site, where TOC values were measured at 0.06% and 0.08%, a sediment volume of 20 g was necessary to obtain a detectable lipid content.^80^ In typical cases where TOC values are not available prior to sampling, the color of archaeological sediments can serve as a rough indicator of organic content. Lipid biomarker studies focusing on PAHs necessitate larger sediment volumes, sometimes reaching up to 150 g, e.g., Brittingham et al.^79^ This is primarily due to the relatively low occurrence of these volatile molecules. However, acquiring such volumes of sediment poses a challenge in archaeological contexts where sedimentary facies representing several decades or centuries may have a thickness of less than 1 cm and be laterally limited to one or two meters (e.g., the sedimentary facies from El Salt Middle Palaeolithic site^182^). The integration of lipid analysis with soil micromorphology is recommended, as it offers the opportunity for microcontextual control over variables such as estimated organic content and sedimentation rates. Lipid analysis ideally should be performed directly on micromorphological samples, and recent method development in this area is yielding promising outcomes. In a study to explore the potential of direct molecular and isotopic lipid biomarker analysis on polyester resin-impregnated sediment slabs from archaeological micromorphology Rodríguez-de Vera et al.^189^ showed positive identification *n*-alkanes, aromatics, *n*-ketones, and alcohols and variability in the representation of fatty acids, which are widely present in polyester resin. The potential to measure δ^13^C C_16:0_ and C_18:0_ was also explored in this study, which reported a significant isotopic shift in resin-impregnated slabs when compared with samples without resin, particularly in the C_18:0_ values.^189^

### Laboratory procedures: Extracting lipids from sediments

Lipid extraction methods depend on the polarity and chemical structure of the target compounds, which vary. In some cases, obtaining a total lipid extract (TLE) for direct injection into a gas chromatographer is sufficient. In others, it is preferable to first separate the TLE into different fractions to isolate specific biomarkers. This is a laborious process that can be time-consuming and demanding, especially when dealing with many sediment samples. First, the sediment samples must be dried (in an oven, through nitrogen freeze drying or lyophilization). McClymont et al.^210^ recommend freeze-drying, as alternative methods may result in the loss of certain compounds. Furthermore, their findings suggest that extraction from wet sediments may be preferable to drying and subsequent extraction with solvent:water mixtures for some polar compounds. Next, samples must be weighed and ground to a powder using an agate mortar and pestle. During this step, meticulous care must be taken to pull out visible organic particles, including bone fragments, charcoal (or other dark particles), and modern rootlets, as their presence can introduce unwanted interference or noise to the sample. These elements are frequently encountered in archaeological sediments. When the goal is to explore biomarkers that might be present in all the different lipid fractions, for each powdered sediment sample the extraction process typically requires: (1) obtaining the total lipid extract (TLE) and (2) isolating different biomarkers from the total lipid extract into fractions of different polarity. The TLE extraction involves the use of organic solvents and solid-liquid extraction methods, followed by phase separation (centrifugation, filtration, etc.).

In general, there is no single protocol for sample preparation that is universally suitable for all archaeological matrices and compatible with all analytical techniques. Various techniques, such as Soxhlet extraction,^211^^,^^212^^,^^213^ microwave-assisted extraction (MAE),^129^^,^^156^^,^^212^^,^^214^^,^^215^ accelerated solvent extraction (ASE, also called pressurized solvent extraction -PLE),^78^^,^^143^^,^^216^ and ultrasonic-assisted extraction (UAE),^90^^,^^211^^,^^217^^,^^218^^,^^219^ are commonly employed to enhance extraction efficiency, reduce time, and minimize solvent volume. Preferred solvents like DCM/MeOH and chloroform/MeOH, in different proportions depending on the target molecules, are commonly used, although acetone and hexane are also employed. Methanol is effective for extracting polar molecules like fatty acids and alcohols, while DCM is suitable for saturated and unsaturated hydrocarbons due to its lower polarity. Lipid matter in archaeological sediments consists of free hydrocarbons, other lipid compounds, and esterified compounds. For esterified compounds, such as bile acids, steroids and fatty acids, which are mainly conjugated, saponification or acidic hydrolysis is necessary. This involves addition of a hydroxide/methanol solution and the relevant internal standards (ISs). Importantly, although *n*-alkanes are typically not esterified, humic acids can retain significant amounts of hydrocarbons,^220^ resulting in less than 10% of the total alkanes and fatty acids extracted. Therefore, besides solvent selection, extraction time is also crucial to achieve higher yields. Extraction times vary significantly depending on the extraction protocol, ranging from 72 h for some Soxhlet methods^211^^,^^221^ to just a few minutes for MAE or ASE.^78^^,^^215^

For investigations focusing on the *n*-alkanes, a QuEChERS-based extraction method proposed by Herrera-Herrera et al.^222^ has shown to obtain similar recovery values while reducing the extraction time and the amount of solvent used (10 mL per extraction from 5 g of sediment) when compared to other methods. The QuEchERS (“Quick, Easy, Cheap, Effective, Rugged, and Safe”) protocol was developed by Anastassiades et al.^223^ to extract pesticides from fruits and vegetables with high water content. It consists of two steps: a solid-liquid extraction with salting-out, and a clean-up step through dispersive solid-phase extraction (dSPE). By reducing the time, this method also reduces the likelihood of autoxidation reactions that may influence the analytes.^222^ However, despite the advantages, its use should be carefully evaluated when samples contain a very low alkane concentrations since, as demonstrated in other research fields, extracts obtained through the QuEChERS method have lower concentrations (1 g/mL) compared to other techniques (2–5 g/mL).^224^

Column chromatography is a common method for separating different compound families from the total lipid extract (TLE). It involves dissolving the analytes in organic solvents, loading them onto a column, and eluting them sequentially with solvents of different polarity. The choice of stationary phase, such as aminopropyl, silica gel, alumina or silver infused sorbents, depends on specific needs. Aminopropyl, a silica-based stationary phase functionalized with amino groups, is effective in retaining acids and separating carboxylic acids from neutral lipid fractions.^211^ Silica, a polar adsorbent, causes non-polar compounds to elute first.^225^ Silver nitrate-infused silica gel is used to separate lipids based on their degree of unsaturation and simplify complex lipid mixtures.^226^ Alumina is ideal for electron-rich compounds, such as GDGTs. Additionally, lipid-rich samples can slow down solvent elution due to partial clogging of the column by the TLE. One potential drawback related to the use of silver nitrate is that the silver ion (Ag^+^) is light-sensitive and can decompose to elemental silver, reducing its efficiency over time.^211^

### Derivatization, saponification and silylation

Alcohols, fatty acids and other polar compounds are not sufficiently volatile and therefore not suitable for analysis using gas chromatography. For this reason, they require their derivatization, a process that enhances the volatility and performance of polar lipids. In this regard, fatty acids are derivatized to the more volatile *trans*-esterified fatty acid methyl esters (FAMEs). From a methodological perspective, there are several approaches that can be employed.^227^ Commonly used catalysts include acidic ones, such as HCI,^179^ H_2_SO_4_,^112^^,^^219^ and BF_3_,^228^ and alkaline ones like NaOCH_3_,^227^ KOH,^118^ NaOH, or diazomethane.^109^ Each catalyst has its distinct characteristics and limitations for specific applications, so careful attention must be given at every step, particularly during the formation of FAMEs and the subsequent recovery processes.^227^ For example, free fatty acids cannot be esterified by alkaline catalysts and require strict anhydrous conditions.

Fecal lipid biomarker analysis requires a somewhat more complex process, due to the typical esterification of sterols and stanols in sediments. First, the TLE must be saponified, as described earlier. Then, if subsequent IRMS analysis is not needed, *silylation*, or conversion of alcohols into trimethylsilyl (TMS) derivatives is carried out. A similar process is applied to a considerable portion of di- and triterpenoid lipids. For this purpose, various derivatization procedures exist,^229^ including commercially available pre-mixed silylation reagents. Popular reagents such as BSTFA (N,O-bis(trimethylsilyl) trifluoroacetamide),^90^^,^^152^^,^^163^ MSTFA (N-methyl-N-trimethylsilylfluoroacetamide),^230^ and HMDS (hexamethyldisilazane)^138^^,^^139^^,^^140^ are often used in combination with catalysts,^229^ such as TMCS (trimethylchlorosilane),^90^^,^^138^^,^^139^^,^^140^^,^^152^^,^^213^ TMIS (trimethyliodosilane),^230^ and/or pyridine.^138^^,^^139^^,^^140^ It’s worth noting that silylation has also been used for the derivatization of fatty acids.^217^^,^^219^ While silylation requires less time than methylation, its products degrade over time. Consequently, silylation is not recommended for CSIA. A current challenge is to identify silylation derivatizing agents with known isotopic compositions.

### Lipid compound detection, quantification, calibration, and characterization

The majority of lipid compounds, being apolar and volatile, can be detected using gas chromatography. However, polar lipid compounds, such as GDGTs or linear ketones, require liquid chromatography. This technique may also be better suited for other polar lipids, including steroids, such as sterols, stanols, and bile acids. Injection and measurement parameters in contemporary gas chromatography-mass spectrometry (GC-MS) are variable and constrained by the capabilities of today’s commercial instruments and the analytes’ specificities. Most of the published work is carried out using GC-MS instrumental setups with hot vaporizing injectors (i.e., split/splitless, programmable temperature vaporizer, multimode) typically programmed in splitless mode (although different split ratios could be needed if samples are very concentrated). Separation time settings can be adjusted according to specific requirements, and in case of very complex samples, it is advisable to use longer durations to prevent compound overlaps. For example, GC separation of alkanes, once isolated from other lipid components through pretreatment and extraction procedures, is notably simpler and quicker compared to separating complex isomeric mixtures of fatty acids or alcohols. The stationary phases of capillary columns depend on molecular polarity. For less polar compounds (such as *n*-alkanes or PAHs) non-polar or relatively low polar phases (100% dimethylpolysiloxane and (5% phenyl)-methylpolysiloxane) are required, whereas polar phases such as cyanopropyl are used for FAMES. Regarding the mobile phase, H_2_, N_2_, Ar, and He have classically been employed as carrier gases. Even though hydrogen provides faster separation, helium is currently the preferred gas because it is inert, can be obtained at high purity, and provides excellent performance.^231^ However, in recent years, helium demand has exceeded the supply and it can be costly and difficult to deliver to some regions.

Detection and measurement of analytes commonly relies on commercially available MS and IRMS, which are rapidly advancing technologies that exhibit enhanced sensitivity. The average sensitivity of commercialized standard MS instruments is currently around 5 μg/L or even lower, while the sensitivity for IRMS instruments depends on the particular isotope analyzed. This advancement is crucial in compound-specific isotope analysis (CSIA) of typically limited archaeological sediment volumes. For instance, conducting hydrogen isotope analysis using a standard sensitivity commercial IRMS instrument necessitates an amount of target analytes unlikely to be present in 5 g of archaeological sediment.^80^^,^^83^ Increasing the volume of sediment samples in archaeological contexts can be challenging. Our objective is to identify specific molecules within the sediment that hold environmental and behavioral significance. In archaeological contexts, these variables may fluctuate within millimeters or centimeters of space, and such spatial distances may also equate to shifts in time. Therefore, analyzing large sediment volumes risks obtaining mixed, diachronous molecular data. Using high-end instruments with increased sensitivity that allow us to analyze small sediment volumes helps mitigate this limitation. Although a similar limitation has been reported in non-archaeological paleoclimate research,^232^^,^^233^ organic matter concentrations in lake sediment samples are generally higher than in mixed archaeological sediment samples, with some exceptions, as indicated by reported TOC values. For instance, TOC values lower than 0.1% were reported in a Middle Paleolithic sequence,^80^ whereas values higher than 27% were observed in a lacustrine sequence.^57^

Climate and archaeological scientists analyzing sedimentary lipids put efforts into quantification and calibration aspects. In the measurement of *n*-alkanes and their δ^2^H and δ^13^C values, interpretations primarily rely on relative values and the relative proportions among distinct lipid molecules. In archaeology, some scientists have used pseudo-quantification through the determination of peak areas (or percentage of peak areas),^27^^,^^114^^,^^133^^,^^150^ or by using rough estimates that involve the inclusion of an IS at a known concentration. Estimation of analyte amounts is then performed based on the proportional area of the IS.^21^^,^^141^^,^^234^^,^^235^^,^^236^ Other researchers apply calibration curves, where the instrumental signal is mathematically related to the analyte concentration and instrumental calibration is performed and the compound concentration is expressed as ng or mg of individual compound or total concentration per gram of dry sample.^79^^,^^80^^,^^90^^,^^107^^,^^128^^,^^179^^,^^198^^,^^214^^,^^237^

A recent study also applies matrix-matched calibration,^237^ which accounts for the “matrix effect”, whereby the sedimentary substrate (usually a complex organo-mineral clayey substrate) may enhance or decrease the matrix-free behavior of lipid molecules and therefore condition the measurement processes. The results of the study indicate that matrix-matched calibration is an optimal approach for stand-alone or mass spectrometric chromatography (GC-MS) of archaeological sediment samples. Matrix-matched calibration involves collecting and extracting control sediment samples from the target site as calibration blanks. From a theoretical standpoint, it is ideal for the matrix-controlled sediment sample to be at least five times larger than the target samples to ensure the accurate generation of calibration curves. However, achieving this ideal is challenging due to the complex, heterogeneous, and often unknown organic and inorganic composition of archaeological deposits. Previous research suggests a significant matrix effect when comparing instrumental and matrix-matched calibration methods, yet there is inconsistency in observing this effect across different soil types.^238^^,^^239^^,^^240^^,^^241^ This indicates that various soils and sediments may yield similar matrix effects, potentially rendering the soil type variable less influential in determining the consistency and magnitude of the matrix effect. Typically, geogenic sources, mineralogy and large-scale geographic variables are homogeneous within archaeological sites. Therefore, when collecting samples for lipid biomarker analysis in archaeological sites, it is recommended to collect a representative control sediment sample weighing around 25–30 g from any stratigraphic profile or surface and any stratigraphic unit. Additional control samples should be collected if the target lipid biomarker samples are from contexts or units with contrasting color and texture, such as when collecting samples from very dark (organic-rich) and light-colored (inorganic) sediment within a single site. Testing the consistency of the matrix effect when comparing the different control samples will inform whether a single matrix-matched calibration can be applied for the entire sample set.

Two additional methodological factors that should be considered in lipid biomarker analysis are: (1) compound identification and (2) the choice of IS. First, identification typically involves relying on characteristic ions and comparing mass spectra with references from the scientific literature and commercially available databases of mass spectra references. While these approaches are acceptable, the only way to ensure accurate identification of a compound is by injecting an authentic standard under the same analytical conditions. This is often impractical due to the high number of biomarkers and their associated costs (or even the lack of commercial standards for some compounds). Second, selecting an appropriate IS is crucial. The ideal IS should have a similar structure and chemical behavior to the target molecule but should not be present in the original sample. Additionally, if analytes undergo a derivatization process, the IS should be capable of undergoing the same derivatization to ensure optimal chromatographic properties (see Table 1 for examples of commonly used IS). For MS analysis, isotopic standards that are identical to the measured analyte, except for the additional stable isotope mass are considered ideal ISs. The amount of IS used should be within a similar concentration range as the analytes being measured. For now, the use of isotopic standards is limited by their commercial availability, which is currently relatively scarce.

Lipid analysis of archaeological sediment samples requires specific considerations for extraction, detection, measurement, and characterization due to several unique challenges. As emphasized throughout this text, the choice of analytical protocol depends on multiple factors: the specific lipid type (e.g., esterified bile acids versus PAHs), the nature of the archaeological context (e.g., highly organic samples from a stable versus highly inorganic ones), the research goals and questions, available technical resources and sample material, and the analyst’s expertise. These factors can lead to significant variation in analytical protocols. An effort in protocol standardization is needed to yield comparable datasets among researchers applying lipid biomarker analysis to archaeological sediments. Only by applying increasingly accurate and precise methods—such as matrix-matched calibration for quantification or standardized definitions for various biomarker ratios—can we achieve unambiguous comparisons, leading to a deeper understanding of the molecular information contained within each sample.

## Outlook and final remarks

Sedimentary lipid biomarkers from archaeological contexts are a valuable source of paleoenvironmental and behavioral information. As the number of geoarchaeological case studies involving sedimentary lipids increases, there are at least three points that stand out.

(1) There is a need for expanding reference data. Food, agriculture, and environmental science is a robust source of reference data on the lipid composition of many different plants, animals and their derivatives and products. However, the apparent geographic variability in isotopic fractionation requires further reference data of plants and animals from different regions and altitudes, to assess archaeological lipid isotopic biomarker data with higher degrees of certainty. There is effort in this direction, as in some of the work mentioned in this paper that includes lipid molecular and isotopic profiling of local endemic vegetation,^89^^,^^242^ as well as other work that includes such valuable reference data for the investigation on lipid residues in archaeological materials.^176^^,^^243^ It is also important to control the effect of diet, particularly when dealing with ancient prehistory, when the diet of animals was likely very different from their modern analogues. Experimental and ethnoarchaeological work is crucial to support archaeological lipid biomarker data focused on biological sourcing to approach different aspects of past human behavior. ^14^^,^^161^^,^^244^^,^^245^^,^^246^^,^^247^
(2) The current research reveals a certain degree of equifinality in lipid degradation caused by different processes (i.e., aerobic, diagenetic, or thermal degradation) and this reality hampers paleoenvironmental and behavioral interpretations. The problem is encountered in sedimentary lipid biomarker research relating to paleoenvironmental reconstruction, biological sourcing, and fire. Although each of these research lines has come up with ways to overcome this issue, there is need for a dedicated line of basic research into the different conditions of lipid molecule preservation and the mechanisms of hydrolytic, aerobic, and thermal degradation that may undergo in different depositional environments, i.e., lipid biomarker taphonomy. As in other archaeological subdisciplines, the study of taphonomic processes requires an assessment of the effect of single and multiple variables on the degradation of organic matter and characterization of the different degradation stages of lipid compounds and their isotopic ratios. This can be achieved through a combination of experiments and deep-time evidence from archaeological case-studies.
(3) The complexity and multi-source nature of archaeological site formation processes is a crucial limiting factor for geochemical sourcing analysis such as sedimentary lipid biomarker analysis. There is great difficulty in discerning between anthropogenic and natural (geogenic or biogenic) organic matter input in time-averaged sediment samples containing complex mixes of well-preserved and partially degraded organic residues from different sources. This requires that archaeological sedimentary lipid biomarker analysis is conducted in combination with other analyses, including ones that can provide proxies for biogenic and anthropogenic input (e.g., ancient DNA, proteomics, phytolith, pollen and non-palynomorph particle analysis, anthracology, carpology, and zooarchaeology) and others that provide clues about the depositional and postdepositional conditions and processes including weathering and chemical and physical transformations by fire or other agents (e.g., magnetic susceptibility, mineralogy, elemental, and other geochemical analyses) from a microcontextual approach that incorporates soil micromorphology as a high resolution spatiotemporal anchor and a visual aid to composition and sedimentary processes in their stratigraphic context.

Studied along with other proxies and through a microcontextual approach, lipid biomarkers provide detail in our interpretation of the human past and fill some of the gaps in the elusive organic archaeological record. The state-of-the-art in lipid biomarker analysis of archaeological sediments has largely resulted from applying concepts and methods used in non-archaeological sediments, which in turn stem from basic geochemistry research. There are recent promising methods yet to be tested in archaeological sediments, such as direct radiocarbon dating of sedimentary lipids^248^^,^^249^^,^^250^^,^^251^^,^^252^^,^^253^ and archaeological pottery.^254^^,^^255^^,^^256^^,^^257^ Successful application of this method will add another milestone to the current bioarchaeological revolution. In turn, archaeological case studies provide a window of opportunity to explore diagenetic processes through time and in different depositional settings, contributing valuable knowledge to geobiologists and geochemists. Continued interdisciplinary feedback will facilitate the mutual advance of all the disciplines that explore lipid biomarkers.

## Acknowledgments

The authors would like to thank the members and collaborators of the AMBILAB for their work toward the development of sedimentary lipid analysis in archaeology, the support of the IUBO-ULL and three anonymous reviewers whose comments and recommendations contributed significantly to the current version of the paper. Some of this work has been carried out with the financial support of an ERC Consolidator grant (ERC-CoG-PALEOCHAR-648871, PI Carolina Mallol), the 10.13039/501100011033Spanish State Research Agency (PID2019-107113RB-I00, PI Carolina Mallol), Fundación Caja Canarias and “La Caixa” (project 2018PATRI19, PI Carolina Mallol and Project IMPACT, 2022CLISA04, PIs Margarita Jambrina-Enríquez and Natalia Égüez), a Marie Skłodowska-Curie Actions Global Postdoc grant (101032608 IBERHUNT, PI Natalia Égüez), a Ramón y Cajal Postdoc programme (RYC2022-036901-I, MCIN/AEI/10.13039/501100011033 and FSE+, PI Natalia Égüez), MICIU/AEI/10.13039/501100011033 (project CCR-CAN TED2021-129695A-I00, PI Margarita Jambrina-Enríquez), and European Union Next Generation/PRTR. - Organismo Autónomo de Parques Nacionales (project PALEOMOL 2915/2022, PI Margarita Jambrina-Enríquez).

## Declaration of interests

The authors declare no competing interests.
